# Supplementary material for: A yeast phenomic model for the influence of Warburg metabolism on genetic buffering of doxorubicin
Source: Cancer Metab. 2019 Oct 23;7:9. doi: 10.1186/s40170-019-0201-3 (PMC6806529; doi:10.1186/s40170-019-0201-3)
Supplement: Supplementary file 9 — Additional file 9. GO term-specific heatmaps for REMc/GTF-enriched clusters. GO term-specific heatmaps for significant GO process terms were generated as described in methods and Figs. 3 and 4. Any related child terms are presented in subsequent pages of the parent file name. GO terms with more than 100 children, with 2 or fewer genes annotated to the term, or a file size over 300KB are not shown. All heatmaps are generated with the same layout (see Figs. 3 and 4). [file 40170_2019_201_MOESM9_ESM.bz2 › Additional_File9_GOTermHeatmaps/Additional_File9_GOTermHeatmap/regulation_of_protein_serine_threonine_phosphatase_activity.pdf]

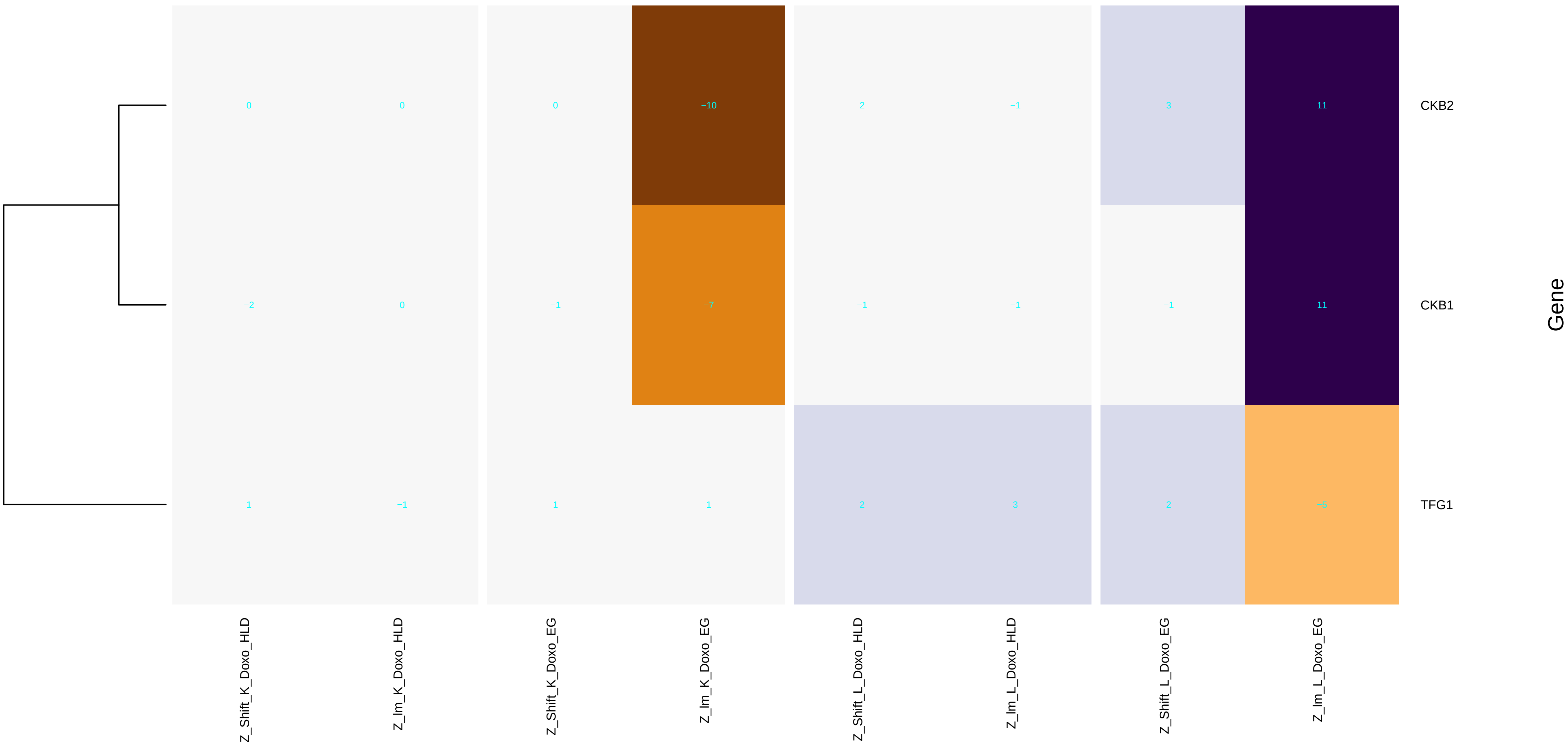

CKB2
CKB1
TFG1
Gene
Z_Shift_K_Doxo_HLD
Z_Im_K_Doxo_HLD
Z_Shift_K_Doxo_EG
Z_Im_K_Doxo_EG
Z_Shift_L_Doxo_HLD
Z_Im_L_Doxo_HLD
Z_Shift_L_Doxo_EG
Z_Im_L_Doxo_EG
0 0 0 −10 2 −1 3 11
−2 0 −1 −7 −1 −1 −1 11
1 −1 1 1 2 3 2 −5
